# Yield of Genetic Testing in Pediatric Cardiomyopathies: Implications for Novel Therapeutic Options

**DOI:** 10.1002/mgg3.70119

**Published:** 2025-07-11

**Authors:** Adelaide Ballerini, Francesca Girolami, Alessia Gozzini, Silvia Passantino, Mattia Zampieri, Alberto Marchi, Alessia Tomberli, Giovanni B. Calabri, Gaia Spaziani, Giulio Porcedda, Elena Bennati, Silvia Favilli, Iacopo Olivotto

**Affiliations:** ^1^ Cardiogenetic Unit Meyer Children's Hospital IRCCS Florence Italy; ^2^ Cardiology Unit Meyer Children's Hospital IRCCS Florence Italy; ^3^ Department of Experimental and Clinical Medicine University of Florence Florence Italy

**Keywords:** emerging treatments, gene therapies, genetic testing, pediatric cardiomyopathies

## Abstract

Pediatric cardiomyopathies are rare, heterogeneous, and challenging conditions, often with a genetic etiology. We estimated the yield of genetic testing in a pediatric cohort with cardiomyopathies and evaluated the potential candidacy to current or emerging treatments based on genetic results. Over one‐third had a conclusive genetic test, including 25% of potential candidates for emerging precision therapies or developing pharmacological options.

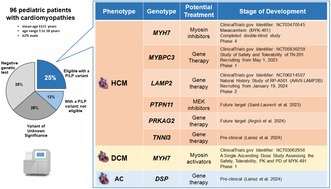

## 1 Introduction

Pediatric cardiomyopathies are rare, heterogeneous, and challenging conditions, often with a genetic etiology, occurring in isolation or as part of complex neuromuscular, metabolic, mitochondrial, or syndromic disease. The four predominant phenotypes are: hypertrophic (HCM), dilated (DCM), arrhythmogenic (AC), and restrictive (RCM) (Lipshultz et al. 2019). Until recently, the utility of genetic testing in cardiomyopathies was limited to confirmation of diagnosis and cascade family screening. Increasingly, however, identifying the genetic etiology has become essential to provide access to novel therapies and to define candidacy for clinical trials and developing targeted treatment options (Parker and Landstrom 2021). Novel approaches including small molecules and gene therapies are emerging and represent the focus of rapidly growing international efforts (Argirò et al. 2024; Lairez et al. 2024; Saint‐Laurent et al. 2023). However, the relevance of these opportunities in contemporary practice remains little appreciated, and genetic testing is still underused. We thus estimated the yield of genetic testing in a pediatric cohort with cardiomyopathies and evaluated the potential candidacy for current or emerging treatments based on genetic results.

## 2 Methods

After genetic counseling, an informed consent was obtained to perfom Next Generation Sequencing (NGS) in a cohort of 96 pediatric patients (mean age 11 ± 5, age range 1–18 years, 62% male) including 44 with HCM, 35 with DCM, 14 with AC and 3 with RCM.

## 3 Results

**FIGURE 1:**
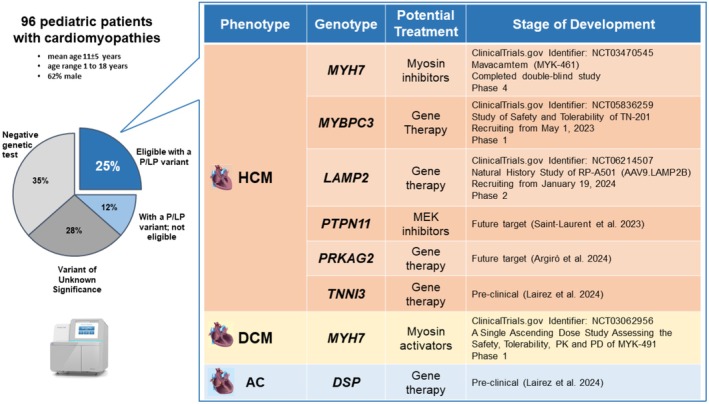
Percentage of patients with a P/LP variant identified eligible to current or emerging treatment. *MYH7*, myosin heavy chain 7; *MYBPC3*, myosin‐binding protein C; *LAMP2*, lysosome‐associated membrane protein 2; *PTPN11*, protein‐tyrosine phosphatase nonreceptor‐type 11; *MEK*, mitogen‐activated protein kinase kinase; *PRKAG2*, protein kinase AMP‐activated noncatalytic gamma‐2; *TNNI3*, troponin I cardiac; *DSP*, desmoplakin.

We identified a pathogenic or likely pathogenic (P/LP) variant in 35 of the 96 patients, that is, a total yield of 37%, while another 34 (35%) had a negative test and 27 (28%) carried variants of unknown significance. Of the 35 patients with P/LP variants, 23 had HCM, 7 had DCM, 3 had AC, and 2 had RCM. The most common etiology was sarcomeric, with *MYH7, MYBPC3*, and *TNNT2* as the most prevalent genes (Figure 1). Additionally, eight were syndromic patients: four with Noonan syndrome (*PTPN11* and biallelic *LZTR1*), two with Danon disease (*LAMP2*), two with Carvajal syndrome (*DSP*), and one with atypical glycogen storage disease (*PRKAG2*).

## 4 Conclusions

In the light of current and emerging treatments, 24 of the 35 gene‐positive patients (68%) were potentially eligible for available or developing pharmacological options including gene therapies. This subset represented 25% (24/96) of the total cohort (Figure 1). In particular, patients with sarcomere gene mutations and HCM might benefit from, or be enrolled in trials with, cardiac myosin inhibitors. The first in class, mavacamten, is already approved for use in adult patients with symptomatic obstructive HCM and aficamten had successfully concluded the Phase 3 study Sequoia; both are being investigated for pediatric use. Conversely, myosin activators are being developed for genetically determined DCM. In addition, clinical studies utilizing MEK inhibitors are focusing on patients with Rasopathies (Saint‐Laurent et al. 2023). Finally, several initiatives are currently targeting gene therapy for cardiomyopathies caused by mutations in *MYBPC3* and *LAMP2* (by gene replacement), while others for TNNI3, *PRKAG2* and DSP‐associated disease are being developed (Argirò et al. 2024; Lairez et al. 2024).

In conclusion, in a consecutive cohort of pediatric patients with cardiomyopathies, over one‐third had a conclusive genetic test, including 25% of potential candidates for emerging precision therapies. In contemporary practice, NGS testing has a key role for diagnosis of pediatric cardiomyopathies, and it is rapidly becoming important for clinical management of a child out of four in the near future.

## Author Contributions

A.B. and F.G. contributed to the design of the study and draft the manuscript; A.B., A.G., and I.O. contributed to analyze the data; S.P., M.Z., A.M., A.T., G.B.C., G.S., G.P., E.B., and S.F. contributed to recruit study participants and collect samples; and I.O. contributed to the design of the study and revise the manuscript. All authors read the final version of the manuscript.

## Disclosure

Prof. Iacopo Olivotto has received grants and personal fees from Bristol Myers Squibb, Cytokinetics, Amicus, Sanofi Genzyme, Shire, Chiesi, Tenaya, Rocket Pharma, Lexeo, and Menarini International.
